# Elevated tumor markers in a benign lung disease

**DOI:** 10.1186/s13019-021-01688-4

**Published:** 2021-10-18

**Authors:** Quan Chen, Qijue Lu, Xiang Fei, Chunguang Li, Bai Li

**Affiliations:** 1 Department of Traditional Chinese Medicine, Changhai Hospital, The Second Military Medical University, No. 168 Changhai Road, Shanghai, 200433 China; 2 Department of Thoracic Surgery, Changhai Hospital, The Second Military Medical University, Shanghai, China

**Keywords:** Pulmonary sequestration, Carbohydrate antigen 19-9, Benign lung disease, Tumor markers

## Abstract

**Background:**

This study aimed to determine the underlying pathophysiologic mechanism of elevated carbohydrate antigen 19-9 (CA19-9) in pulmonary sequestration (PS) patients.

**Materials and methods:**

Four pulmonary sequestration patients, 12 pneumonia patients and 12 healthy adult volunteers were prospectively studied. Specimens from another 34 pulmonary sequestration patients were retrospectively analyzed. Serum CA19-9 levels of 4 patients were tested before and 1 week, 1 month and 3 months after surgery. The CA19-9 levels of 12 pneumonia patients and 12 healthy adult volunteers were tested as controls. The expression and localization of CA19-9 in diseased lesions and corresponding normal lung tissues were analyzed by Immunohistochemical (IHC). Hematoxylin–eosin (HE) staining was performed to observe the pathological changes in pulmonary sequestration tissues.

**Results:**

Serum CA19-9 levels were significantly higher in the 4 patients (797.3 ± 316 IU/ml) than in the pneumonia patients (10.07 ± 5.01 IU/ml) and healthy volunteers (9.85 ± 4.12 IU/ml). In addition, serum CA19-9 levels decreased dramatically after the focus was removed. Positive staining of CA19-9 was found in 70% (24/34) of pulmonary sequestration tissues, and CA19-9 was mainly expressed in the bronchial mucus. In the 4 diseased lesions, deformed alveolar structure and inflammatory cell infiltration were observed, and the degree of damage was positively correlated with serum CA19-9 levels.

**Conclusions:**

CA19-9 could be generated by abnormal columnar epithelia in pulmonary sequestration tissues and was transported into circulation after alveoli damage. CA19-9 could serve as an adjuvant diagnostic marker in pulmonary sequestration.

## Introduction

**Fig. 1:**
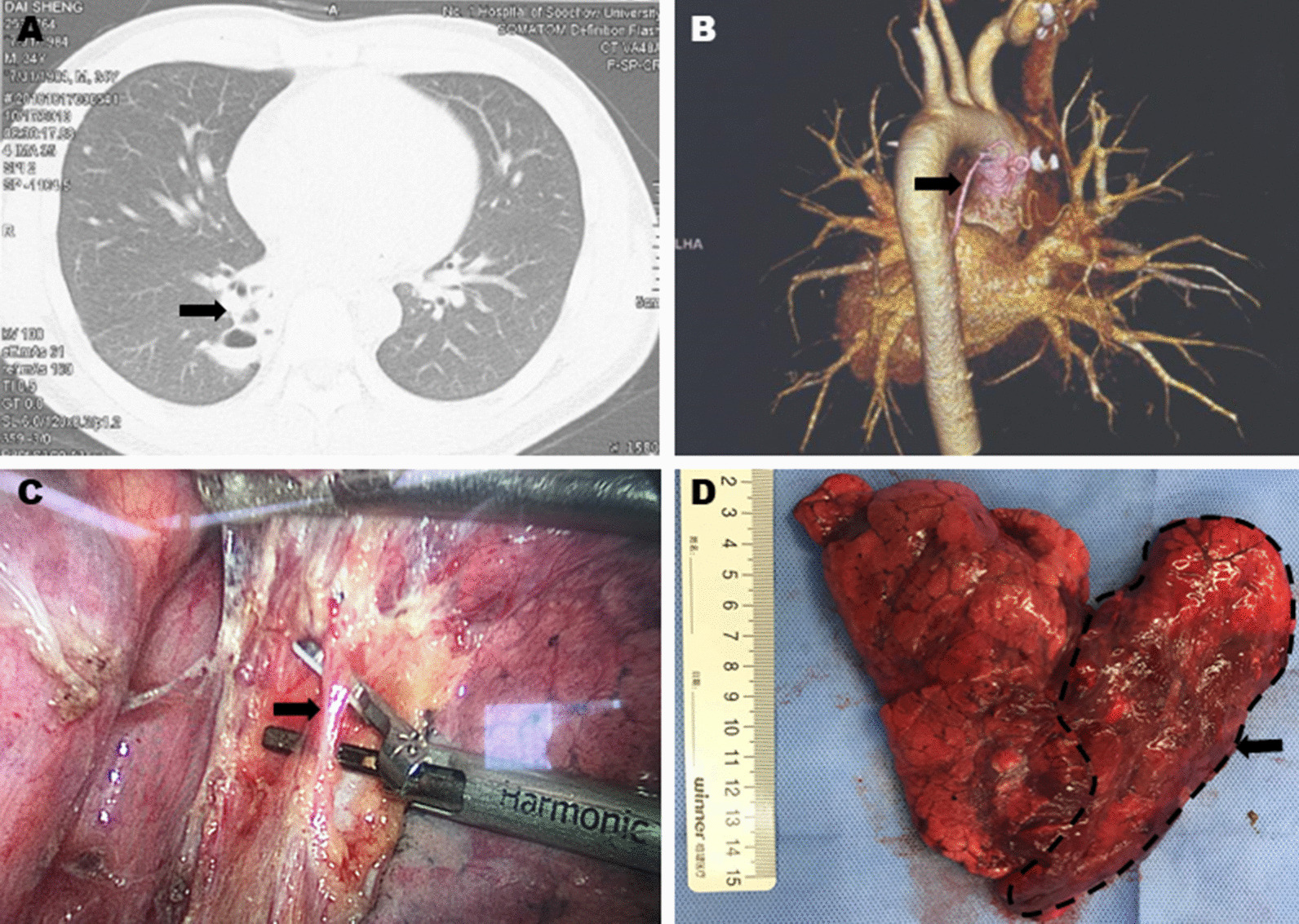
Intra-pulmonary sequestration. **A** CT scan of the PS lesion in right lower lobe (black arrow). **B** Aberrant supply artery originated from descend aorta in 3D reconstruction (black arrow). **C** The supply artery in operation (black arrow). **D** Pathological specimen of right lower lobe

Pulmonary sequestration accounts for 0.15–6.4% of all congenital pulmonary malformations and receives its blood supply from a systemic artery rather than the pulmonary arterial branch [1–5]. The predominant clinical symptoms of pulmonary sequestration consist of coughing (50%), fever (25%), hemoptysis (22.7%), expectoration (18.2%), chest tightness (15.9%) and thoracic pain (11.4%) [6]. Diagnosis is mainly based on chest CT to find local focal opacity, especially in the left lung (Fig. 1A). Confirmation of aberrant supply arteries usually relies on aortography or contrast-enhanced chest CT (Fig. 1B) [7]. The management of PS includes surgical resection (Fig. 1C, D) and aberrant artery embolization, but differences in prognoses mainly depends on the presence of accompanying malformations and perioperative complications [8].

Elevated serum levels of CA19-9 commonly indicates malignancies in the digestive tract, biliary tract, pancreas, or lungs [9, 10]. CA19-9 is observed in the normal epithelial lining of the biliary tract, gastric mucosa, pancreatic duct, and bronchial glands [11]. A small number of patients were admitted to the hospital with continuously elevated serum CA19-9. Radiologic examination found lung malformations, and pathological results verified the diagnosis of PS. These findings drove us to explore the relationship between PS and CA19-9 and the underlying mechanism.

## Methods

### Group processing

Serum CA19-9 levels in 12 pneumonia patients, 12 healthy adult volunteers and 4 PS patients in the prospective group were detected prior to surgery and compared. In addition, the cystic fluid from the 4 PS patients was collected and tested for CA19-9 expression, and the serum CA19-9 levels of the 4 PS patients was monitored continuously in the follow-up after surgical resection. The tissue specimens from 4 PS patients in the prospective group and 34 PS patients in the retrospective group were acquired for H&E staining and IHC staining.

### Patient identification and sample collection

Two asymptomatic female patients of 29 and 44 years of age were admitted to our department for abnormally elevated CA19-9 (665 IU/ml and 458 IU/ml, respectively). The medical examinations of both patients found a focus with high intake of 18-FDG in the left lower lobe and right lower lobe, respectively. A supply artery originated from the descending aorta in iterative reconstruction. Another 54-year-old female came to the hospital with intermittent fever and cough in addition to the increased level of CA19-9 (1200 IU/ml) in the last 6 months. Enhanced contrast CT detected a mass in the left S10, and the nourishing vessel originated from the descending aorta. A 31-year-old male complained of continuous cough and shortness of breath and exhibited a high level of CA19-9 (866 IU/ml). A chest CT scan showed a mass in the left lower lobe. All 4 patients were diagnosed with pulmonary sequestration, 3 of whom received a minimally invasive lobectomy, and the pathologic results confirmed the PS diagnosis. In addition, we drained fluid from the cyst lumen with a 5 ml injector after the specimen was resected, and the supernatant was collected to detect CA19-9 levels after centrifugation at 1500 r/min for 5 min. Furthermore, we collected 5 ml blood from 12 pneumonia patients and 12 healthy adult volunteers to measure the CA19-9 level for comparison. The criteria of pneumonia were as follows: (a) had fever, cough, expectoration and other aspiration symptoms; (b) CT scan or X-ray confirmed the thickened lung texture and inflammatory exudation; and (c) elevated WBC, neutrophil and C-reactive protein levels. The paraffin-embedded tissues of 34 pathologically diagnosed PS patients from 2010 to 2018 in our hospital were kindly offered by the pathology department (Table 1). All patients received lobectomy or wedge resection with VATS. The study was approved by the Ethics Committee of Changhai Hospital, the Second Military Medical University. Signed informed consent was obtained from all the participants.

**Table 1:** Characteristics and tumor markers status of 34 patients

General features	NO.	Tumor markers	NO.	Clinical symptoms	NO.
Gender		CEA		Coughing	
Male	20	Elevated	2	Positive	18
Female	14	Normal	7	Negative	16
Age		Unknown	25	Fever	
≥ 40	15	CA19-9		Positive	10
< 40	19	Elevated	3	Negative	24
Location of lesion		Normal	5	Hemoptysis	
Left lower	24	Unknown	26	Positive	6
Right lower	9	CA125		Negative	28
Right upper	1	Elevated	2	Expectoration	
Feeding artery		Normal	4	Positive	8
Thoracic aorta	28	Unknown	28	Negative	26
Abdominal aorta	3	CA242		Chest tightness	
Coeliac trunk	1	Elevated	1	Positive	4
Bronchial artery	1	Normal	1	Negative	30
Intercostal artery	1	Unknown	32	Thoracic pain	
				Positive	5
				Negative	29

### Immunohistochemical staining

Paraffin-embedded tissues, including infected and noninfected lung portions from the 34 patients, were serially sectioned into 5-µm thickness slices. These samples were deparaffinized and rehydrated. Epitope retrieval was performed in heated citrate buffer for 15 min, cooled and immersed in 3% hydrogen peroxide for 30 min to block endogenous peroxidase activity. Nonspecific binding sites were blocked by incubation with 5% goat serum (Gibco, 16210064) for 30 min. These sections were then incubated with a primary mouse anti-human antibody against CA19-9 overnight at 4 °C. A biotinylated goat anti-mouse secondary antibody was added for 30 min, followed by incubation with avidin–biotin-peroxidase complex for another 30 min. CA19-9 immunoreactivity was calculated by evaluating the percentage of positively stained cells.

### Enzyme-linked immunosorbent assay

Briefly, 5 ml cyst fluid or blood was collected and centrifuged at 1500 r/min for 5 min to acquire the supernatant. CA19-9 was assessed via commercially available ELISA kits according to the manufacturer’s instructions. The absorbance was read at 450 nm on a FL600 Microplate Reader, and all the samples were analyzed in duplicate.

### Morphological observations

To visualize the morphology of alveoli and the pathological changes caused by PS, both normal and focus tissues were collected and fixed in 10% formaldehyde at 4 °C overnight. The samples were embedded in paraffin and sliced into 5-μm-thick sections for hematoxylin and eosin staining (H&E). The integrity of alveoli and inflammation infiltration were observed and photographed under an optical microscope.

## Results

### CA19-9 was upregulated in the tissues and peripheral blood of PS patients

**Fig. 2:**
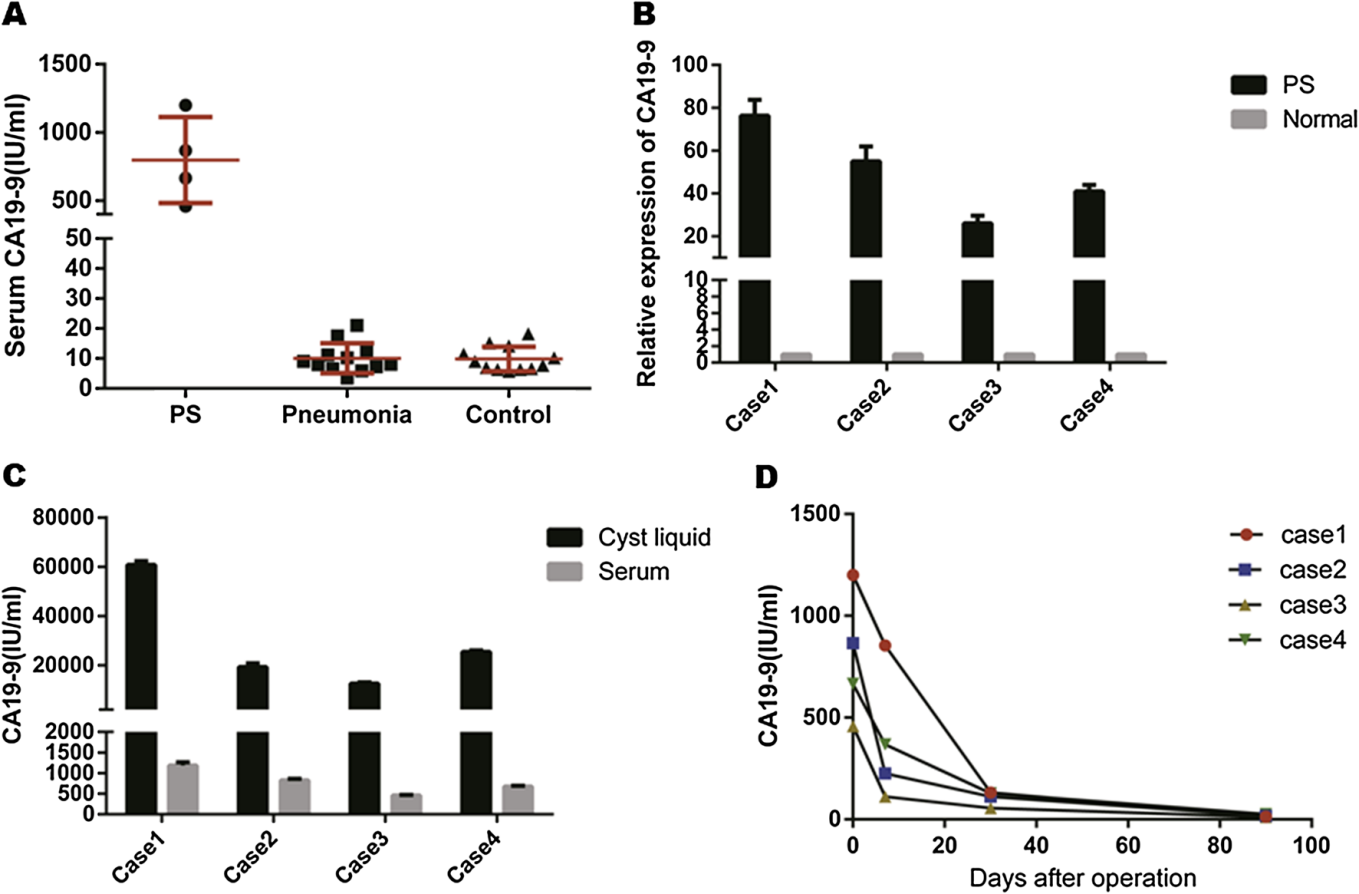
**A** Serum CA19-9 in PS patients, pneumonia patients and healthy controls. **B** Relative expression of CA19-9 in diseased and the corresponding normal lungs. **C** Comparison of CA19-9 between serum and cyst fluid in the 4 patients. **D** Serum CA19-9 of the 4 patients before and 7 days, 30 days and 90 days post operation

**Fig. 3:**
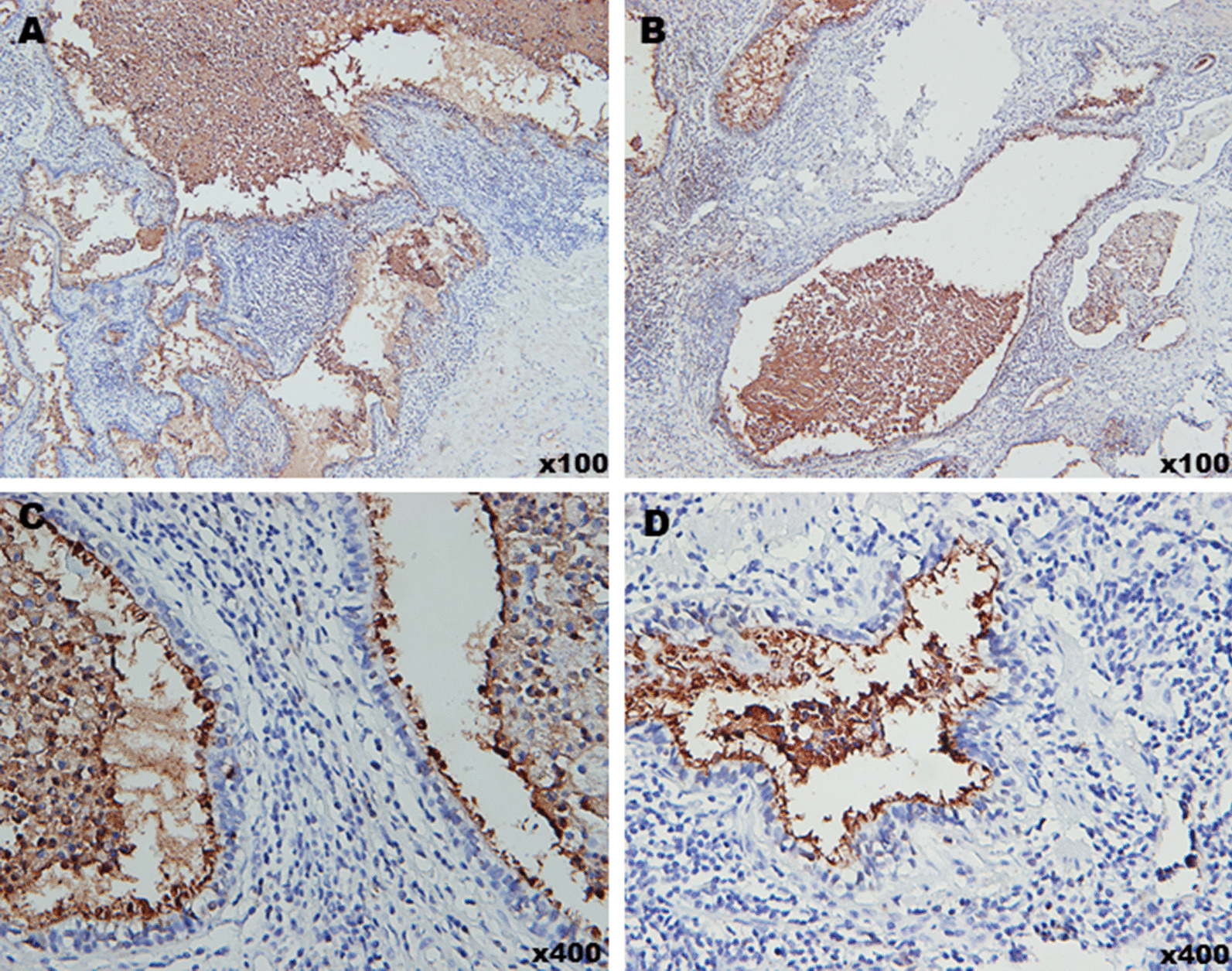
IHC staining of CA19-9 of PS tissues: CA19-9 was strongly expressed in PS tissues and mainly in bronchial mucus and alveolar space. **A**–**D** Represented the expression of CA19-9 in specimen from the 4 patients with a serum CA19-9 of 1200 IU/ml, 866 IU/ml, 665 IU/ml and 458 IU/ml, respectively

Serum CA19-9 levels of the first 4 patients was abnormally upregulated (797.3 ± 316 IU/ml), but neither the pneumonia patients (10.07 ± 5.01 IU/ml) nor the healthy volunteers (9.85 ± 4.12 IU/ml) had elevated serum CA19-9 levels (Fig. 2A). Furthermore, the CA19-9 levels in diseased lungs were significantly higher than that in normal lungs (Fig. 2B). IHC staining of 34 PS specimens revealed that CA19-9 was strongly expressed in 24 lesions compared to their adjacent normal lung tissues. In addition, the positively stained areas were mainly observed in the bronchial mucus and alveolar space, which indicates that they are produced in the columnar epithelia of respiratory glands (Fig. 3). There was no evidence of malignancy in any of the resected specimens.

### CA19-9 was highly expressed in the cystic fluid of PS tissues

After centrifugation, the supernatant from 5 ml cyst fluid was carefully collected to determine the expression of CA19-9 and to confirm that CA19-9 could be secreted into the alveolar lumen by columnar epithelia of respiratory glands. ELISA results verified that the CA19-9 levels in cyst fluid reached 60,129 IU/ml, 21,056 IU/ml, 12,665 IU/ml and 25,608 IU/ml, and the corresponding serum CA19-9 levels were 1200 IU/ml, 866 IU/ml, 458 IU/ml and 665 IU/ml, respectively (Fig. 2C).

### The expression of CA19-9 in PS patients was correlated with the degree of alveolar damage

**Fig. 4:**
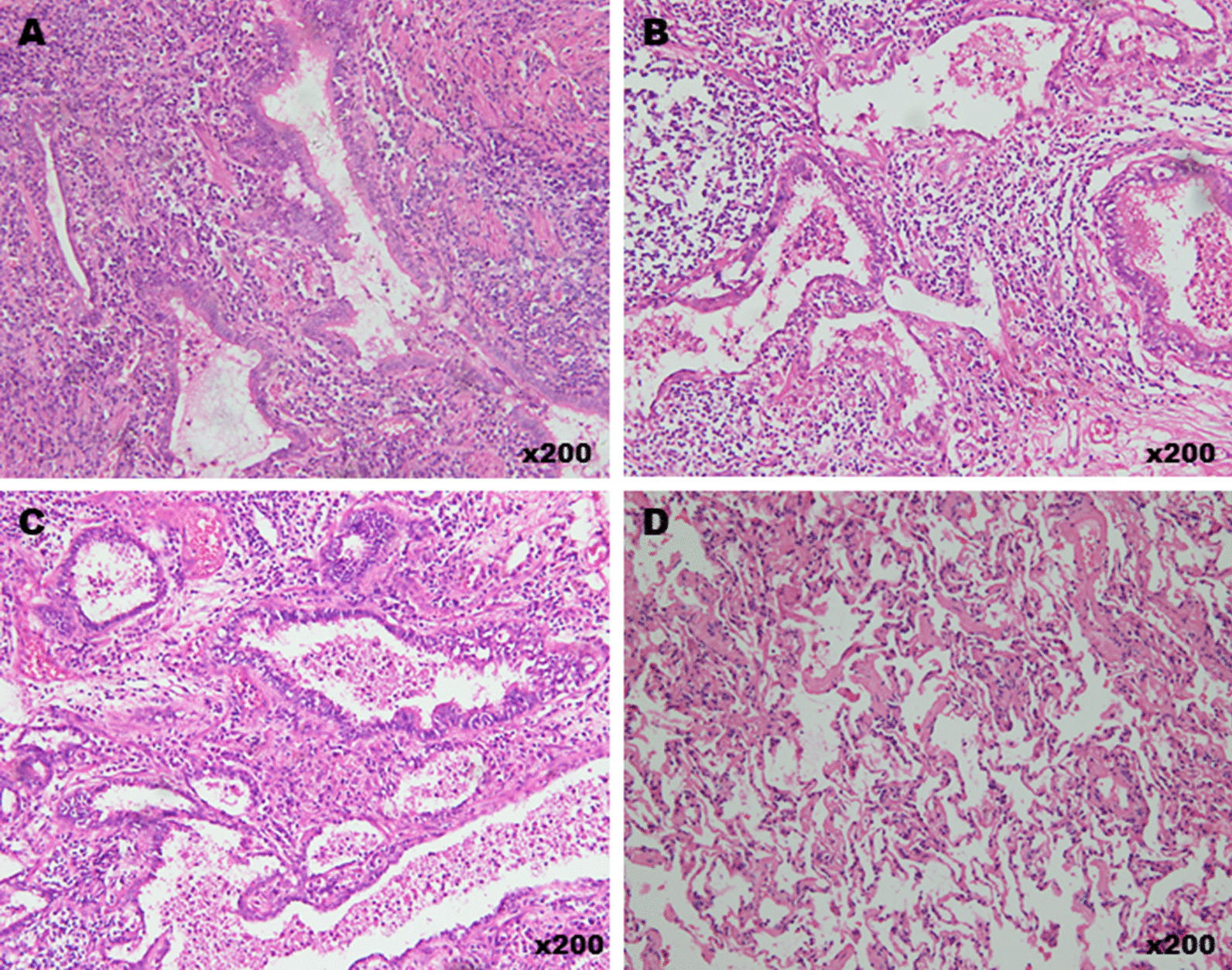
H&E staining of PS tissues from 4 different patients carried elevated CA19-9. **A** In the patient with CA19-9 of 1200 IU/ml, the alveoli walls were severely crushed and the alveolar space filled with inflammatory cells. **B**, **C** A moderate degree of inflammation was found in the specimen of the 2 patients with serum CA19-9 of 866 IU/ml and 665 IU/ml, respectively. **D** In the patient who has a CA19-9 of 458 IU/ml, the normal alveolar structure could be observed and less inflammatory cells in alveolar space and septum

The CA19-9 produced in the epithelia of the sequestration tissue may have become concentrated in the mucus in the cysts and then transferred into the blood through the injured mucosa of the cyst walls. PS patients frequently suffer from pneumonia, and inflammatory cells can break the cyst walls and invade into the alveolar space. Thus, it is reasonable to evaluate the degree of alveolar damage according to inflammatory cell infiltration and alveolar morphology. H&E staining of the 4 PS tissues provided a visualization of inflammatory cell infiltration and alveoli morphology. In the patient with 1200 IU/ml CA19-9, we found that the alveolar walls were severely crushed and that the alveolar space was filled with inflammatory cells (Fig. 4A). In the patient who had a CA19-9 level of 458 IU/ml, a normal alveolar structure was observed, and fewer inflammatory cells were observed in the alveolar space and septum (Fig. 4D). A moderate degree of inflammation was found in the specimens of the other 2 patients with serum CA19-9 levels of 866 IU/ml and 665 IU/ml (Fig. 4B, C).

### CA19-9 levels decreased after the PS was resected

To further validate our hypothesis that CA19-9 was generated from aberrant lesions, we determined CA19-9 expression in peripheral blood at 7 days, 30 days and 90 days after resection and focused on the change in CA19-9 levels in the follow-up. As illustrated in Fig. 2D, the levels of CA19-9 in all 4 patients decreased over time and returned to normal 3 months after the lesion was removed.

## Discussion

PS is a segment of the lung parenchyma that is separated from the tracheobronchial tree and receives its blood supply from a systemic artery rather than a pulmonary arterial branch [1–3]. The diagnosis of PS requires focal opacification and a systemic arterial supply, which is usually verified by contrast-enhanced CT or aortography [7]. The management of PS includes surgical resection and conservative therapy for asymptomatic patients [8]. CA19-9 is an important and well-recognized tumor marker. Elevated serum CA19-9 levels are significant because they may indicate malignancies in the digestive tract, biliary tract, pancreas, or lungs. CA19-9 expression is observed in the normal epithelial lining of the biliary tract, gastric mucosa, pancreatic duct, and bronchial glands [9–11]. Before our study started, we treated two patients who complained about continuously elevated CA19-9 levels, which is recognized as a tumor marker in the digestive tract, biliary tract, pancreas, or lungs [10], and radiographic examination found no abnormalities other than a focus in the lung. The pathologic diagnosis after surgery was PS, which is a benign disease. These findings motivated us to determine the relationship between CA19-9 and PS.

Previous studies from Komatsu H et al. indicated that the bronchial mucus contains large amounts of CA19-9, which appears to be produced in the columnar epithelia of respiratory glands, even if the serum CA19-9 levels are normal [12]. The CA19-9 produced in the epithelia of the sequestration tissue may have become concentrated in the mucus in the cysts and then transferred into the blood through the injured mucosa of the cyst walls [10].

When we reviewed 34 PS patients, we surprisingly noticed that CEA, CA125 and CA242 were increased in 2, 1 and 1 cases, respectively (Table 1). Dewan and Pelosi proposed that the columnar epithelia within PS tissue may maintain many characteristics of embryonic stem cells to some extent [13, 14]. These findings explain why tumor markers, including CA19-9, CEA, CA125 and CA242, that are generally secreted by embryonic stem cells, were upregulated in PS patients. Since we considered PS to be a benign lung disease, tumor markers were not routinely tested after patients were admitted, and we suspected that more PS patients carried elevated tumor markers. We recently received an email from a 40-year-old female whose CA19-9 level bas been over 400 IU/ml since 2013, and a CT scan revealed a mass in the right lower lobe. She consulted some studies and worried about the possibility of lung cancer and even waived the idea of pregnancy. She was released after being told that the elevated CA19-9 might be caused by pulmonary sequestration and is now prepared to receive surgery.

CA19-9 and other tumor markers are not specific for cancer; therefore, to avoid potential diagnostic pitfalls, it is important for clinicians to be aware of respiratory diseases associated with elevated serum levels of these markers. To determine the mechanism underlying CA19-9 generation and the corresponding signal transportation, further, more in-depth studies are required.

## Conclusion

Our study demonstrated that CA19-9 levels in PS tissues were significantly higher than that in normal lung tissues. CA19-9 levels were increased in the peripheral blood of PS patients, especially those with a pneumonia history, and the expression was positively correlated with the severity of pneumonia. We concluded that CA19-9 may be produced by the columnar epithelia of respiratory glands, and it accumulates in the bronchial mucus in cysts of the dysplastic region. When the mucosa of the cyst walls is injured, CA19-9 could be transferred to the peripheral blood and then detected. The concentration of CA19-9 in the cyst was much higher than that in blood. After the mass was surgically removed, CA19-9 levels decreased to normal in a short period and have not increased thus far.

## Data Availability

Bai Li is the corresponding author who could be contacted and data are available upon reasonable request.

## Abbreviations

PS: Pulmonary sequestration
CA19-9: Carbohydrate antigen 19-9
IHC: Immunohistochemical
HE: Hematoxylin–eosin
VATS: Video-assisted thoracic surgery
Elisa: Enzyme linked immunosorbent assay
CEA: Carcinoembryonic antigen
CA125: Carbohydrate antigen 125
CA242: Carbohydrate antigen 242
